# Complete remission of Cdx-2 positive primary testicular carcinoid tumor: 10-years follow-up and literature review

**DOI:** 10.1186/s12894-020-00768-2

**Published:** 2020-12-14

**Authors:** Eugen Widmeier, Hannah Füllgraf, Cornelius F. Waller

**Affiliations:** 1 Department of Medicine IV, Medical Center – University of Freiburg, Faculty of Medicine, University of Freiburg, Freiburg, Germany; 2 Institute of Surgical Pathology, Faculty of Medicine, University of Freiburg, Freiburg, Germany; 3 Department of Medicine I, Medical Center – University of Freiburg, Faculty of Medicine, University of Freiburg, Hugstetter Strasse 55, 79106 Freiburg, Germany

**Keywords:** Neuroendocrine tumor, Primary testicular carcinoid tumor, Histopathology, Biomarkers, Cdx-2 positivity, Metastatic tumor of unknown origin, Case report

## Abstract

**Background:**

The neuroendocrine cells can cause a variety of malignancies throughout the human body known as the neuroendocrine tumors (NETs) or carcinoid tumors. The primary testicular carcinoid tumor (PTCT) accounts for less than 1% of the testicular neoplasms and for only 0.2% of all carcinoid tumors representing already a very rare neoplastic entity. Here, we present a patient with a history of an exceptionally rare primary testicular carcinoid tumor, staining positive for Cdx-2 along with a literature review.

**Case presentation:**

A 44-year old patient without significant past medical history was diagnosed in September 2009 with primary testicular carcinoid tumor, which was surprisingly staining positively for Cdx-2, too. At the time of the initial diagnosis the tumor was already showing histopathological infiltration of veins. DOTA-TATE-PET/CT imaging and endoscopy studies did not show any signs of distant metastases and in particular no gastrointestinal manifestation following no further medical indication for systemic chemotherapy. The continuous and close follow-up of the patient has reached a total of over 10 years at the time of publication remaining in complete remission.

**Conclusion:**

The diagnosis of primary testicular carcinoid is based on histopathology. The detailed histopathologic assessment of biomarkers based on immunohistochemistry is very important for the classification and the prognosis of the primary testicular carcinoid tumor. Primary testicular carcinoid tumor with Cdx-2 positive stain outlines an exceptionally rare neoplastic entity without a consensus about general follow-up guidelines, requiring close clinical and imaging aftercare and consideration in Cdx-2 positive metastatic tumor of unknown origin.

## Background

The neuroendocrine cells can cause a variety of malignancies throughout the human body known as the neuroendocrine tumors (NETs). These types of tumors, likewise called carcinoid tumors, are able to produce hormone-like molecules, which can mimic characteristic hormonal syndromes [1]. Carcinoid tumors occur predominantly in the gastrointestinal tract (67.5%) and in the bronchopulmonary system (25.3%) [2]. The primary testicular carcinoid tumor (PTCT) accounts for less than 1% of the testicular neoplasms [3] and for only 0.2% of all carcinoid tumors representing a very rare neoplastic entity [4, 5]. The distinction of the origin in primary or metastatic circumstance is of the outmost importance for the prognosis of the testicular carcinoid tumor and the treatment [6]. Here, we present a case history of a primary testicular carcinoid tumor with exceptionally rare Cdx-2 positive stain and complete remission along with 10 years recurrence-free follow-up and a literature review.

## Case presentation

**Fig. 1:**
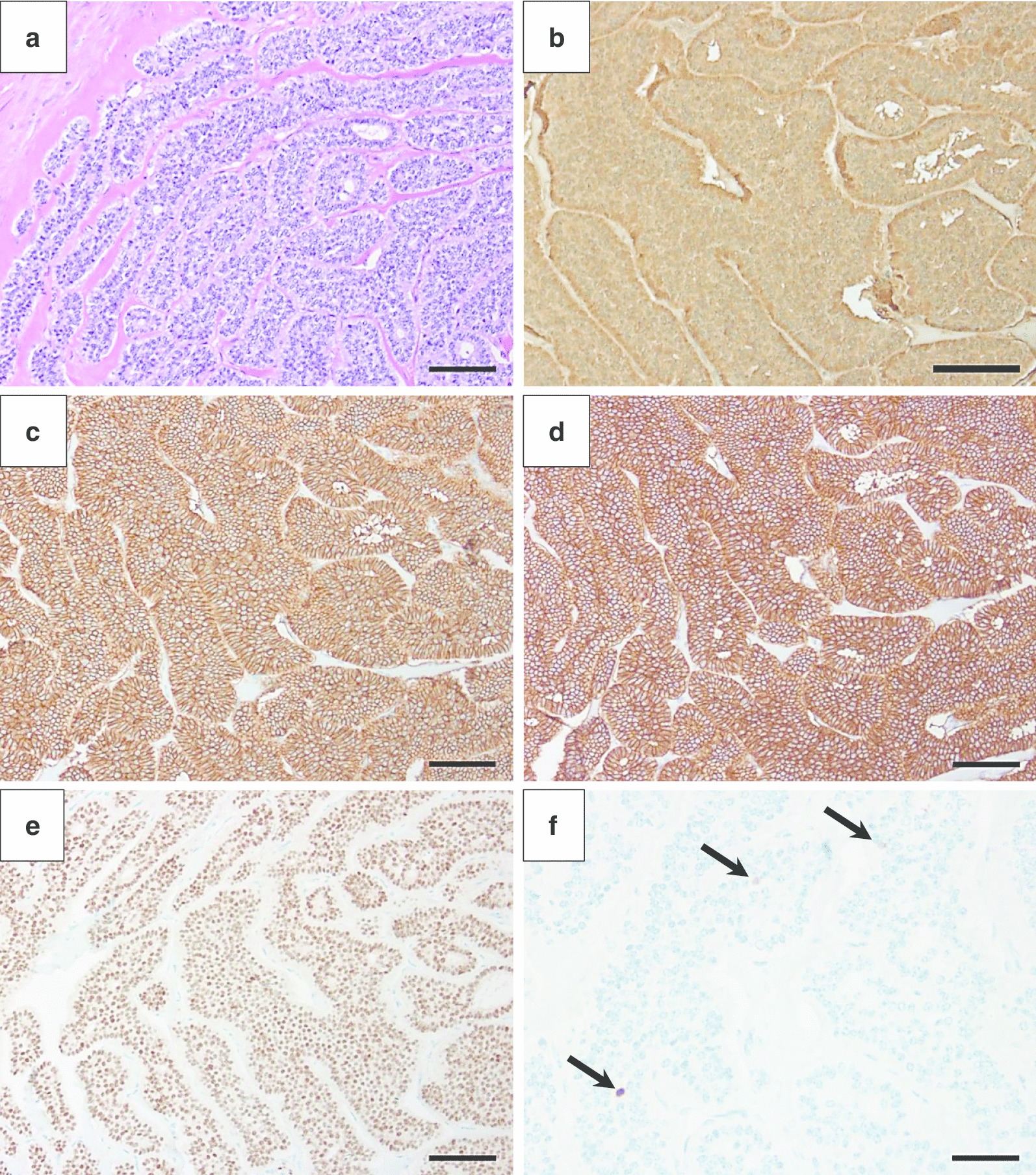
Histological and immunohistochemical analysis of primary testicular carcinoid tumor tissue. Histological analysis (H&E staining) shows nested cuboidal cells with prominent round nuclei and reduced amount of cytoplasm, arranged in a ribbon-like structure and surrounded by moderate fibrotic stroma (**a**), with a positive immunohistochemical staining for Chromogranin A (**b**), CD 56 (**c**), Ep-4 (**d**), Cdx-2 (**e**) and Mib-1(**f**) pointed out by arrows. Scale bars 100 µm

**Fig. 2:**
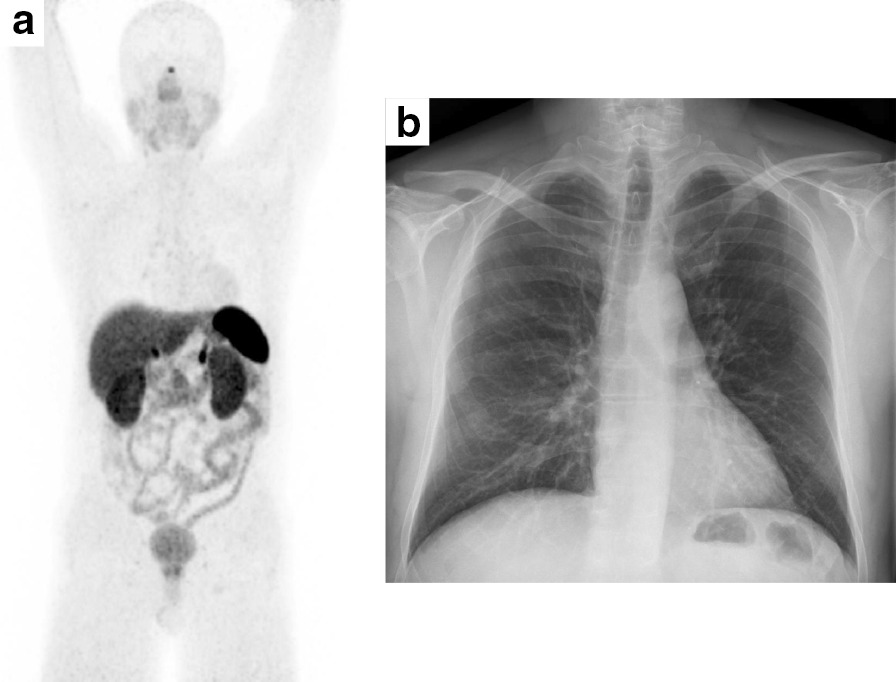
Imaging studies in the context of aftercare. Imaging studies show no signs of primary testicular carcinoid tumor recurrence 3 years DOTA-TATE-PET/CT (**a**) and 10 years Chest X-ray (**b**) after initial diagnosis

We report a 44-year old patient, who saw his urologist in September 2009 due to a painless and for approximately 6 month continuously growing left testicular swelling. The patient did not report any further clinical signs or symptoms of general disease especially no history of sudden hot flushes. The past medical history reveals solely strumectomy due to Grave’s disease and atopic diathesis. On clinical exam no additional noticeable problems aside the one-sided testicular mass, especially no inguinal lymphadenopathy, were detected. The complete blood count and metabolic panel studies were all in normal range. Due to suspected testicular malignancy on clinical exam the patient underwent a near-time radical inguinal left-sided orchiectomy. The histological analysis of the testicular tissue revealed the diagnosis of a primary testicular carcinoid tumor, which was positive for chromogranin A, CD56, Ep4 and Cdx-2. The nuclear MIB-1 expression was detected in patches of the tumor cells with an MIB-1 index of approximately 2% (Fig. 1). At the time of the initial diagnosis the tumor was already showing histopathological infiltration of veins (cN0, cM0, R0 and V1). However the 24-h urine study showed a normal level of 5-Hydroxyindolacetic acid. Based on these findings the patient subsequently underwent DOTA-TATE-PET/CT imaging and endoscopy studies showing no signs of distant metastases and in particular no gastrointestinal manifestation following no further medical indication for systemic chemotherapy. Furthermore the patient was undergoing continuous clinical, abdominal sonography and imaging postsurgical care (Fig. 2). The follow-up of the patient has reached a total of over 10 years at the time of publication remaining in complete remission.

## Discussion

Historically, the nomenclature of “carcinoid” was describing the tumor entity of neuroendocrine cells with low malignancy in the gastrointestinal tract (GI) [7]. However the histopathogenesis of the primary testicular carcinoid remains under debate suggesting respective hypothesis: originating from the same precursor cell as the Leydig cells do or resulting from chromosomal irregularity [8, 9]. The diagnosis of primary testicular carcinoid is based on histopathology. The detailed histopathologic assessment is very important for the classification and the prognosis of the primary testicular carcinoid tumor [3]. Mitotic activity and vascular or tunica albuginea invasion were found not to increase the malignancy of the carcinoid tumors. However the occurrence of a low-degree of tumor differentiation, symptoms of a carcinoid syndrome as well as a tumor size correlate with increased metastatic potential [10, 11]. A precise detection of biomarkers assessed by immunohistochemistry (IHC) enables the subtyping and accurate classification of PTCT [12]. In general it is considered and it was described by Abbosh et al. that caudal type homeobox 2 (Cdx-2), a marker for neuroendocrine tumor cells originating from GI tract, is negative in PTCT [9, 13]. Lee et al. and Bing et al. described malignant germ cell tumors of the testes stained positive for Cdx-2 suggesting precaution in making a diagnosis in metastatic tumor with unknown origin and thereby considering an occult testicular malignancy rather than of GI origin [14, 15]. Albeit Atalay et al. demonstrated a strong significant Cdx-2 staining, as it is observed in tumor cells with GI origin, merely in testicular teratoma [16]. To the best of our knowledge, there is in the literature only one reported case of Cdx-2 positve PTCT [17]. In our case, the exceptionally rare Cdx-2 stain positivity was found as well, albeit no GI origin of carcinoid tumor cells was detected. This data suggests that the analytic specificity of Cdx-2 marker needs to be assessed critically and distinct in each case. The standard treatment of PTCT remains the radical inguinal orchiectomy [6]. The recurrence of PTCT may occur up to 17 years after the initial diagnosis and treatment requiring close clinical and imaging aftercare [18]. Since the data is based on single cases there is no consensus about general follow-up guidelines [6, 19]. In summary PTCT has a good prognosis with the 5-year overall survival rate of 78.7% and the 5-year specific survival rate of 84.3% and should be alternatively considered in Cdx-2 positive metastatic tumor of unknown origin [19].

## Conclusion

Primary testicular carcinoid tumor with Cdx-2 positive stain outlines an exceptionally rare neoplastic entity. The standard treatment of PTCT remains the radical inguinal orchiectomy. PTCT have a good prognosis albeit some prognostic factors increase metastatic potential. The recurrence can appear almost 2 decades after initial diagnosis requiring close clinical and imaging aftercare.

## Data Availability

Not applicable.

## Abbreviations

CD56: Cluster of differentiation 56
Cdx-2: Caudal type homeobox 2
DOTA-TATE-PET/CT: Tetraazacyclododecanetetraacetic acid–DPhe1-Tyr3-octreotate positron emission tomography/computed tomography
Ep4: Prostaglandin E receptor 4
GI: Gastrointestinal tract
IHC: Immunohistochemistry
MIB-1: Marker of proliferation Ki-67
NET: Neuroendocrine tumor
PTCT: Primary testicular carcinoid tumor
